# Taxonomic revision on the genus *Abbottina* Jordan & Fowler, 1903 (Cypriniformes, Gobionidae)

**DOI:** 10.3897/zookeys.1279.185128

**Published:** 2026-05-05

**Authors:** Zhixian Sun, Dong Sheng, Shuya Sun, Wenqiao Tang, Yahui Zhao

**Affiliations:** 1 State Key Laboratory of Animal Biodiversity Conservation and Integrated Pest Management, Institute of Zoology, Chinese Academy of Sciences, Beijing, 100101, China State Key Laboratory of Animal Biodiversity Conservation and Integrated Pest Management, Institute of Zoology, Chinese Academy of Sciences Beijing China; 2 Shanghai Universities Key Laboratory of Marine Animal Taxonomy and Evolution, Shanghai Ocean University, Shanghai, 201306, China Shanghai Universities Key Laboratory of Marine Animal Taxonomy and Evolution, Shanghai Ocean University Shanghai China

**Keywords:** Air-bladder, distribution, East Asia, freshwater fish, morphology, new combination, taxonomy

## Abstract

The genus *Abbottina* Jordan & Fowler, 1903 comprises small benthic gobionid fishes widely distributed in East Asia. However, due to the unclear generic characteristics as defined in the original description, the taxonomic status of the species within this genus remains controversial, and its definition needs clarification. This study examined the morphology of the type species, *Abbottina rivularis* (Basilewsky, 1855), to more accurately redefine generic characteristics. The genus *Abbottina* can be distinguished from all other gobionid genera by the following characteristics: mouth inferior; lips thick, relatively smooth, with small less-developed papillae or shallow grooves; lower lip with anterior fold, medial pad forming two oval protuberances; jaws covered by lips, without horny margin; barbel one pair; the last unbranched dorsal-fin ray thin and soft; with five branched anal-fin rays; pharyngeal teeth in one row; air-bladder moderately large, possessing two soft chambers; anterior chamber enclosed in membrane capsule. Based on morphological characters and phylogeny of mitochondrial genome sequences, *Abbottina lalinensis* has been reassigned to the genus *Microphysogobio*. Further, based on the morphology of *Abbottina binhi* from Cao Bàng Province, this species is reassigned to the genus *Oriengobio*. Thus, the genus *Abbottina* becomes a monotypic genus.

## Introduction

*Abbottina*, a gobionid genus, was established by Jordan and Fowler (1903) based on specimens of *Abbottina psegma* collected from the Yodo River in Osaka, Japan, and was treated as a junior synonym of *Gobio rivularis* Basilewsky, 1855 by Okada (1961). The species in this genus are small freshwater benthic dwellers that inhibit rivers, mountainous streams, and lakes. This genus, commonly seen in East Asia, has a relatively broad distribution ranging from eastern Russia to northern Vietnam (Nguyen and Ngô 2002; Antonov et al. 2019). *Abbottina rivularis*, in particular, is also considered a typical invasive species in many regions (e.g., upper and middle reaches of the Mekong River basin, upper Irtysh and the Ulungur rivers; Neely et al. 2008; Tian et al. 2023).

Since the establishment of the genus *Abbottina*, several species have been described under it (e.g., Luo et al. 1977; Huang and Li 1987). Some of the species were then considered not to belong to *Abbottina* in subsequent taxonomic revisions, which included related genera (Yue 1998; He et al. 2017). The definition of the genus *Abbottina* was vague. One relevant consideration is that the diagnostic characteristics of the genus, as originally outlined by Jordan and Fowler (1903), are not always adequate to clearly distinguish *Abbottina* from closely related genera. Although the latest study tried to revise the genus *Abbottina* phylogenetically (He et al. 2017), a clear definition of the genus is still needed.

Sun et al. (2025) carried out a generic revision on the *Biwia*-*Microphysogobio* complex, which comprises seven genera and is phylogenetically closely related to, but excludes, the genus *Abbottina*. The generic characteristics of each genus within the complex were defined. Consequently, several species formerly assigned to *Abbottina* (Bănărescu and Nalbant 1973; Yue 1998), such as *Biwia springeri* Bănărescu & Nalbant, 1973 (=*Microphysogobio springeri*) and *Pseudogobio obtusirostris* Wu & Wang, 1931 (=*Oriengobio obtusirostris*), have been revised and are no longer retained within *Abbottina* (Sun et al. 2025). Currently, there are three species considered valid within this genus *Abbottina*, i.e., *A. rivularis*, *A. lalinensis* Huang & Li, 1987, and *A. binhi* Nguyen, 2002 (Zhang et al. 2020; Fricke et al. 2025). In order to give a clear definition for the genus *Abbottina*, this study examined the morphology of its type species, *A. rivularis*, and clarified detailed generic characteristics.

## Materials and methods

### Specimen collection and preservation

Examined specimens were collected by hand net and fish trap. Specimens used for morphological study were fixed in 10% formalin solution for 3 days, followed by 70% ethanol alcohol for long-term preservation. Specimens for molecular study were directly fixed in 95% ethanol alcohol. The vouchers were deposited at the Institute of Zoology, Chinese Academy of Sciences, Beijing, China (ASIZB). The information of examined specimens is listed in the section of Materials examined.

### Morphological study

**Figure 1.**
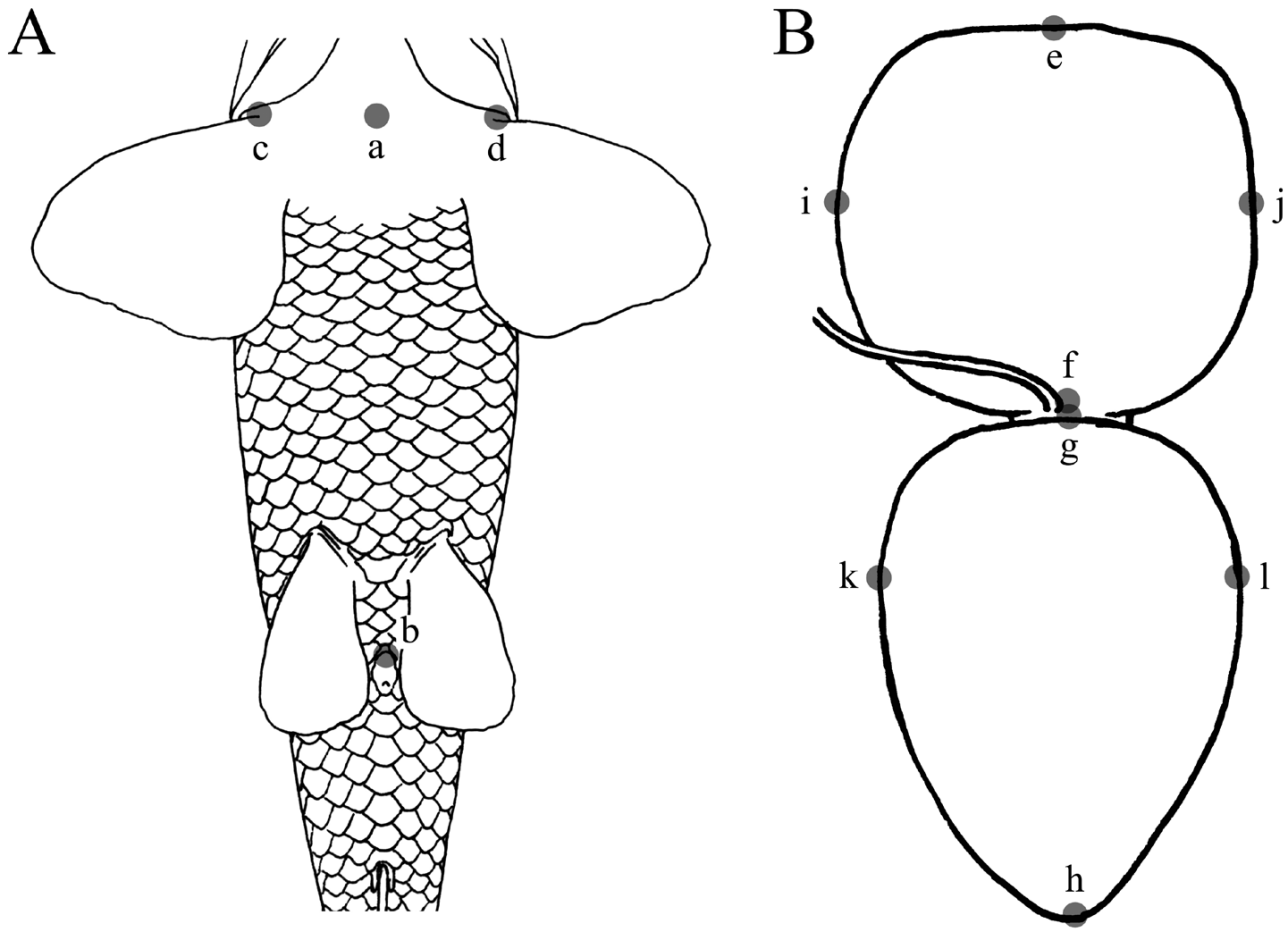
Illustration of morphometric measurements for air-bladder comparison: **A**. Thorax-anal distance and thorax width; **B**. Length and width of air-bladder chambers. The illustration of the abdomen and air-bladder is based on *Abbottina rivularis*, both from a ventral view.

Measurements were taken point-to-point using a digital caliper from the left side of the fish following Sun et al. (2021) for external morphology. The mouth structure was examined and photographed. In order to illustrate the infraorbital bones and lateral ethmoid, the skin and soft tissue on the head of the specimens were carefully cleaned by fine tweezers under a stereomicroscope to expose the margin of the bones. Photographs were made, and illustrations of the infraorbital bones and lateral ethmoid were drawn accordingly. Comparisons on the air-bladder were made, and the measurements on the air-bladder are shown in Fig. 1.

#### Abbreviations

**Thorax-anal distance (a-b)**: the distance from the mid-point of pectoral-fin insertions to the anterior margin of the anus. **Thorax width (c-d)**: the distance between two pectoral-fin insertions. **Total air-bladder length (e-h)**: the distance from the proximal margin of the anterior chamber to the distal margin of the posterior chamber. **Anterior chamber length (e-f)**: the distance from the proximal margin of the anterior chamber to the distal margin. **Anterior chamber width (i-j)**: the maximum horizontal width of the anterior chamber. **Posterior chamber length (g-h)**: the distance from the proximal margin of the posterior chamber to the distal margin. **Posterior chamber width (k-l)**: the maximum horizontal width of the posterior chamber.

### Molecular study

Molecular phylogenetic analysis was conducted based on whole mitochondrial genome sequences. The DNA of *Abbottina lalinensis* was extracted from the pelvic fin on the right side of the specimen using SDS-based extraction combined with a purification column method. The library preparation and genomic DNA sequencing were performed at Beijing TSINGKE Biotech Co., Ltd (China). The sequencing depth was 6×, and approximately 6 Gb of raw data were generated. The preparation and annotation of the mitochondrial genome sequence of *A. lalinensis* follows Sun et al. (2025). A total of 32 sequences, including *A. lalinensis*, were available for phylogenetic analysis. Detailed information on the mitochondrial genome sequences is provided in Suppl. material 1.

Nucleotide sequence alignment was conducted with MAFFT ver. 7.526 (Katoh and Standley 2013). The dataset length after alignment was 16,680 bp. The maximum likelihood (ML) tree was reconstructed using IQ-TREE ver. 3.0.1 (Nguyen et al. 2015) under the model automatically selected by IQ-TREE (GTR+F+I+G4) for 10000 ultrafast (Minh et al. 2013) bootstraps. ModelFinder ver. 3.0.1 (Kalyaanamoorthy et al. 2017) was used to select the best-fit model using the BIC criterion for MrBayes ver. 3.2.7a. Bayesian inference (BI) trees were inferred using MrBayes ver. 3.2.7a (Ronquist et al. 2012) under the GTR+F+I+G4 model (2 parallel runs, 100,000 generations), in which the initial 25% of sampled data were discarded as burn-in. Trees were finally visualized with TVBOT (https://www.chiplot.online/tvbot.html; Xie et al. 2023).

## Results

### Taxonomic account

#### Abbottina

Taxon classificationAnimaliaRotaliidaNonionidae

Jordan & Fowler, 1903

DBC4CD0B-396C-5A55-BF14-BFADC0D72803

**Figure 2.**
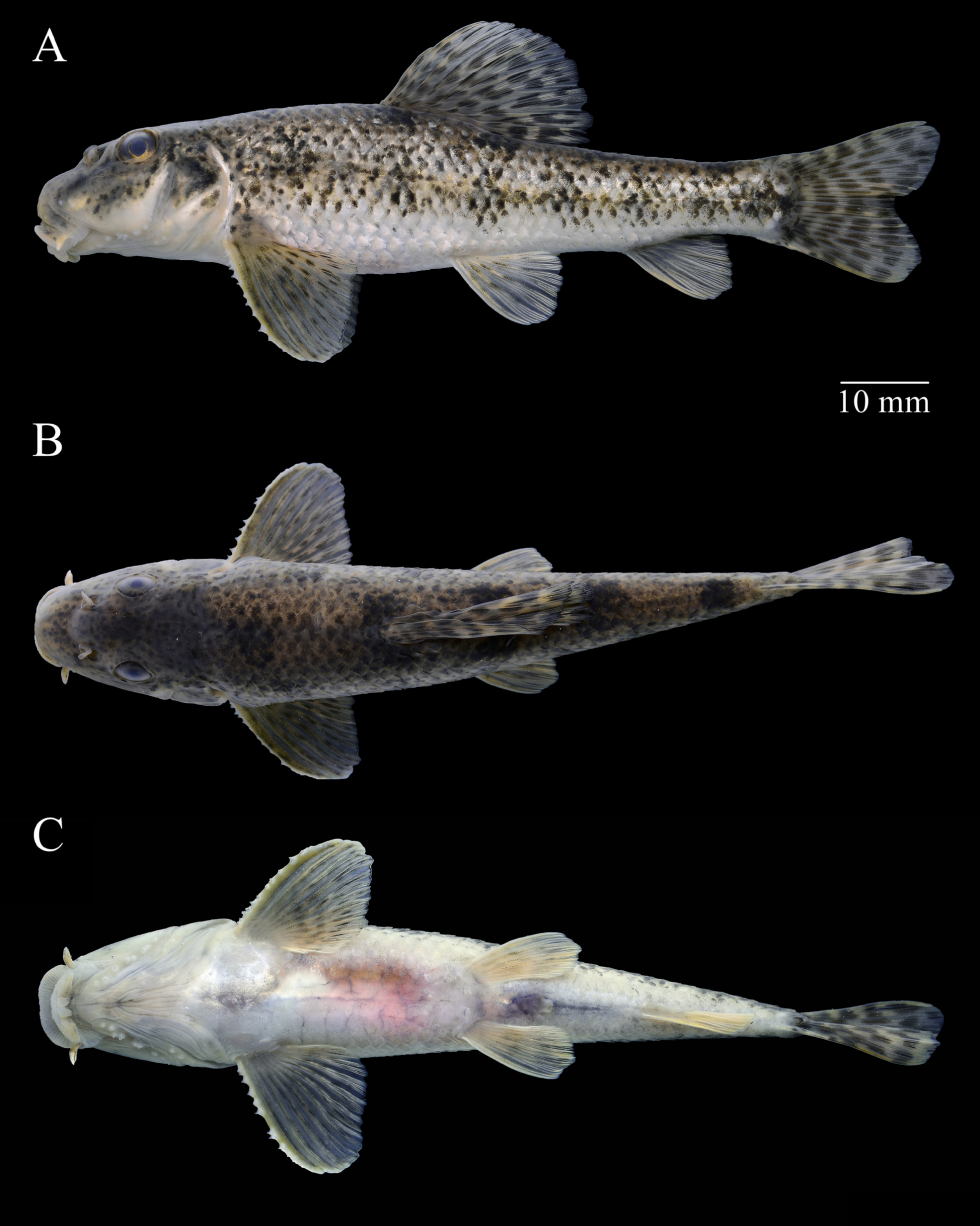
*Abbottina rivularis*, ASIZB 248569, male, 79.3 mm of standard length. **A**. Lateral view; **B**. Dorsal view; **C**. Ventral view. Specimen collected from Liulimiao Township, Huairou District, Beijing, a tributary belonging to the Haihe River basin.

Abbottina Jordan & Fowler, 1903: 835. Type species: Abbottina psegma Jordan & Fowler, 1903, by monotypy.
Pseudogobiops Berg, 1914: 499 (as a subgenus of Pseudogobio Bleeker, 1860). Type species: Gobio rivularis Basilewsky, 1855 (appearance of the type species is shown in Fig. 2).

##### Diagnosis.

**Figure 3.**
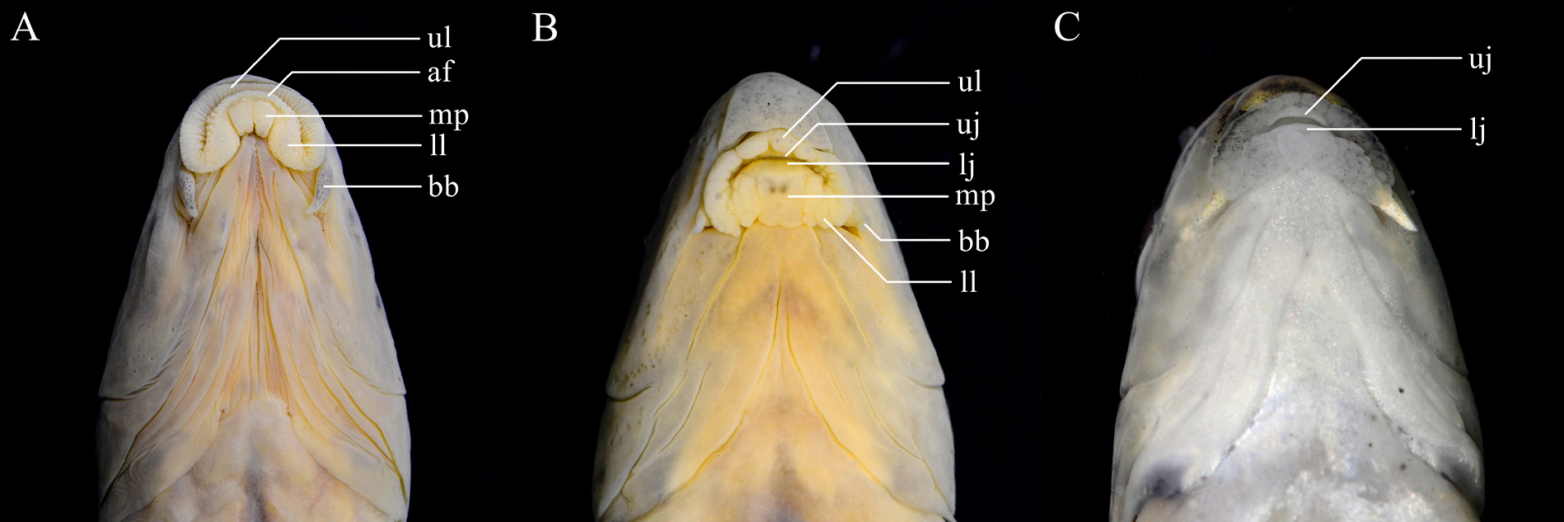
Mouths of *Abbottina* and *Microphysogobio* species: **A**. *A. rivularis*, ASIZB 186016, 66.9 mm SL; **B**. *M. hsinglungshanensis*, ASIZB 240788, 51.7 mm SL; **C**. *M. lalinensis*, ASIZB 248012, 57.8 mm SL. af = anterior fold; bb = barbel, lj = lower jaw, ll = lateral lobe on lower lip, mp = medial pad, uj = upper jaw, ul = upper lip.

**Figure 4.**
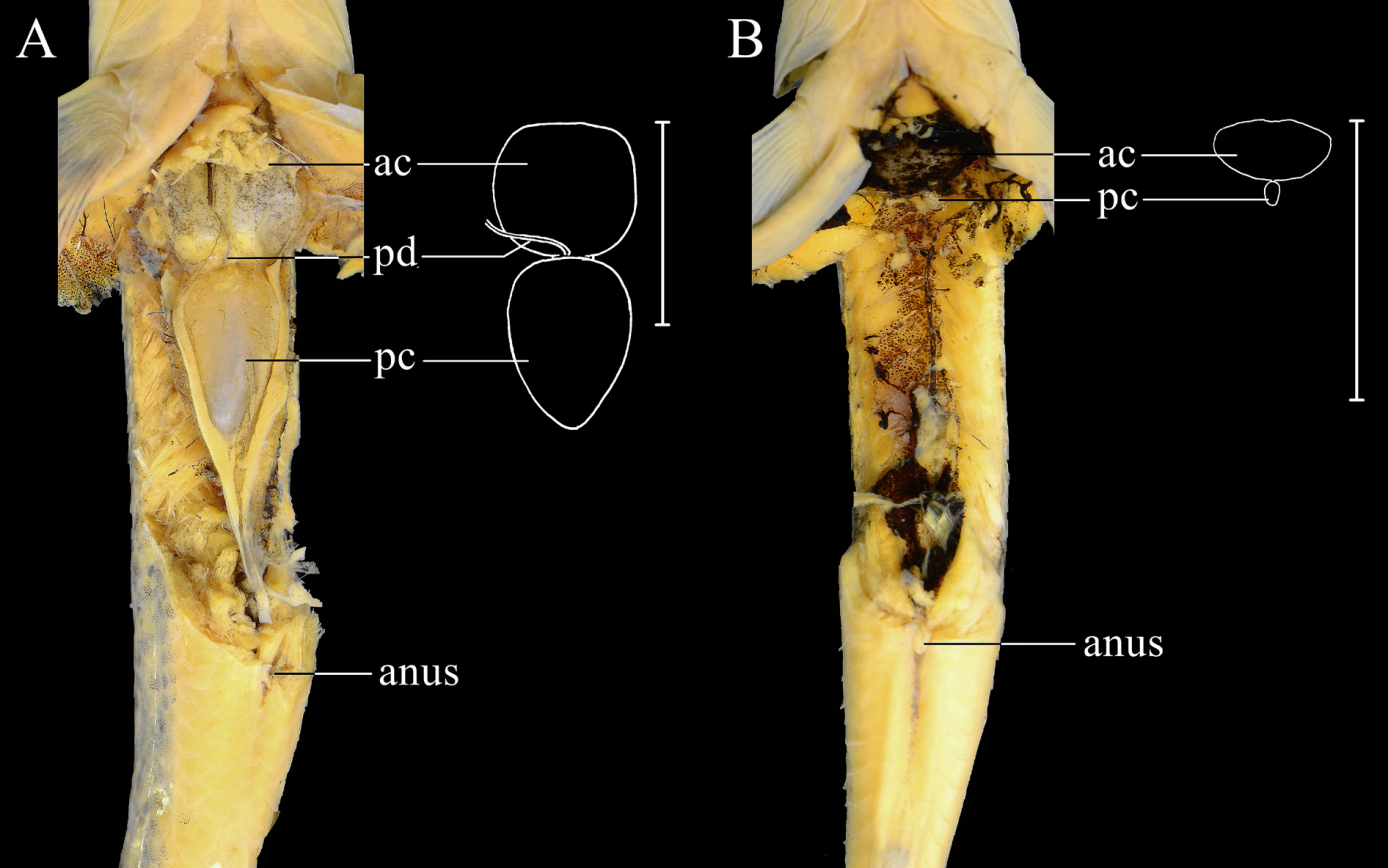
Outline illustrations of the air bladders of *Abbottina* and *Microphysogobio*: **A**. *A. rivularis*, ASIZB 186017, male, 63.6 mm SL; **B**. *M. hsinglungshanensis*, ASIZB 247902, male, 48.3 mm SL. ac = anterior chamber; pc = posterior chamber; pd = pneumatic duct; scales represent 10 mm.

The genus *Abbottina* can be distinguished from all other genera in the family Gobionidae by the combination of the following characteristic: mouth inferior; lips thick, relatively smooth, with small less-developed papillae or shallow grooves; lower lip with anterior fold, medial pad on lower lip forming two oval-shaped fleshy protuberances; jaws concealed beneath lips, without horny margin; barbel one pair (Fig. 3A). The last unbranched dorsal-fin ray thin and soft; anal fin with five branched rays. Pharyngeal teeth in one row. Air-bladder moderately large, with two soft chambers (Fig. 4A).

##### Description.

**Figure 5.**
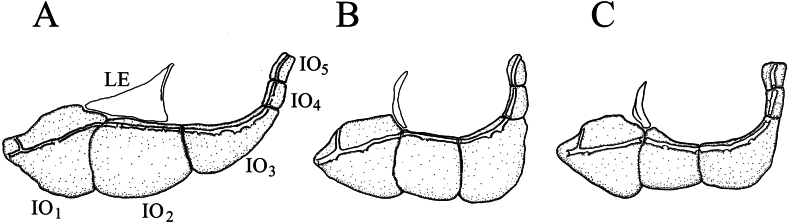
Infraorbital bones and lateral ethmoid of *Abbottina* and *Microphysogobio* species: **A**. *A. rivularis*; **B**. *M. hsinglungshanensis*; **C**. *M. lalinensis*. IO_1_ = 1^st^ infraorbital (lachrymal); IO_2_ = 2^nd^ infraorbital (jugal); IO_3_ = 3^rd^ infraorbital; IO_4_ = 4^th^ infraorbital; IO_5_ = 5^th^ infraorbital; LE = lateral ethmoid.

Body elongated, oblong, abdomen rounded; caudal peduncle short, compressed laterally. Lateral ethmoid broad, triangular in shape. Lachrymal and jugal superiorly contacted with lateral ethmoid, junction length on dorsal margin of jugal three-quarters in total length of dorsal margin, the third infraorbital narrow than the jugal (Fig. 5A). Mouth inferior, lips thick, relatively smooth, with small less-developed papillae or shallow grooves; lower lip possessing one medial pad and two lateral lobes, the medial pad forming two oval-shaped fleshy protuberances, two lateral lobes connected with each other anterior from the medial lobe, forming the anterior fold. Jaws concealed beneath lips, without horny sheathed edge. Body covered with moderately small cycloid scales. Lateral line complete, almost straight. Breast scaleless, ventral region covered with scales. Anus slightly anteriorly positioned from the mid-point of pelvic-fin insertion and anal-fin base. Lateral-line scales 36–39; scales above lateral line 5.5–6.5; scales below lateral line 3–4.5; predorsal scales 11–14, small and irregularly arranged; circumpeduncular scales 12–16. Dorsal fin with seven branched rays, pectoral fin with 10–12 branched rays, pelvic fin with six or seven branched rays, anal fin with five branched rays. The last unbranched dorsal-fin ray thin and soft. Pharyngeal teeth “5–5,” in one row. Air-bladder moderately large, total length slightly larger than half of thorax-anal distance, processing two chambers; anterior chamber rounded, encapsulated in thin soft membrane; posterior chamber larger than anterior chamber, oval-shaped (Table 1). Dorsal and caudal fins with several narrow dark stripe.

**Table 1.** Morphometric measurements on the air-bladder of *Abbottina rivularis*

*Abbottina rivularis* (*N* = 17)			
	Mean	Range	SD
Standard length (mm)	54.5	30.7–83.8	
Thorax-anal distance (TAD, mm)	20.7	11.3–34.4	
In percentage of TAD %			
Total air-bladder length	57.6	48.1–68.0	6.4
Anterior chamber length	23	16.3–29.8	3.4
Posterior chamber length	33.2	23.7–39.4	4.4
Thorax width (TW, mm)	8.6	4.5–13.6	
In percentage of TW %			
Anterior chamber width	63.8	48.0–78.8	8.2
Posterior chamber width	45.7	31.5–57.5	7.8

##### Distribution.

**Figure 6.**
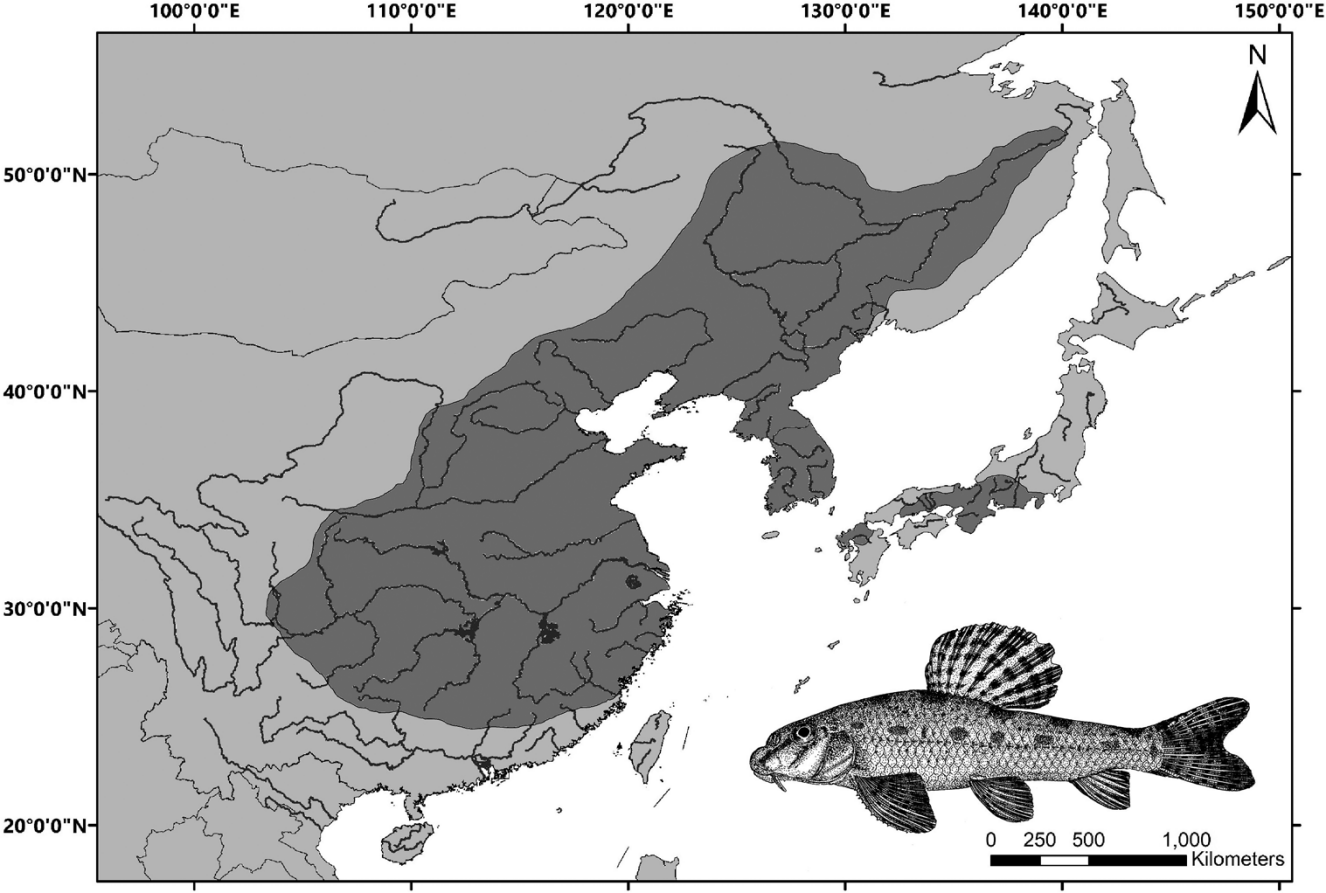
The natural distribution of *Abbottina rivularis* as shown by the dark shading.

This genus is naturally distributed in East Asia, including Russia, Japan, Korea, and China (Fig. 6). The northernmost distribution is the Heilongjiang (Amur) River basin, and the southernmost distribution is the north tributaries of the Pearl River basin, including upstream of the Dongjiang, Beijiang, and Guijiang rivers (Lin 1981; Jin 1990). In the Yellow River basin, it is naturally distributed downstream of Hequ County of Shanxi Province (Li 1981). In the Yangtze River basin, it is naturally distributed to the east of Chengdu Plain, including Chengdu Plain (Zhang et al. 2019; Guo et al. 2021). In Japan, it is only naturally distributed in Honshu and Kyushu Islands (Jang-Liaw et al. 2019).

### Molecular phylogeny

**Figure 7.**
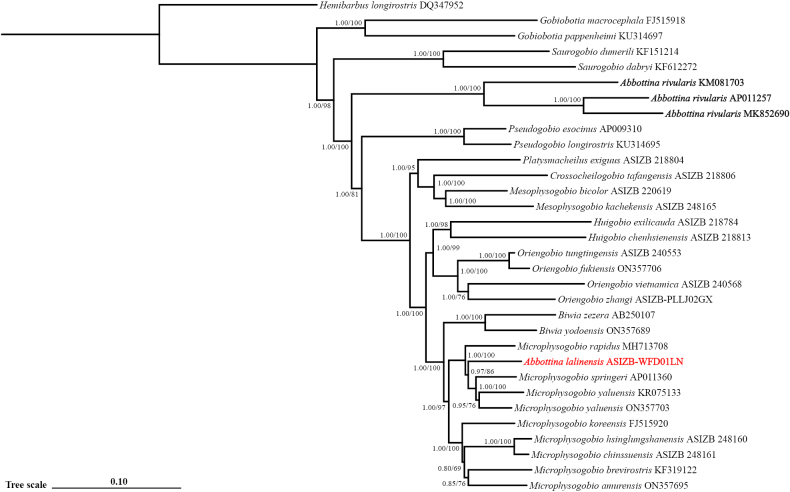
Phylogenetic tree of the genus *Abbottina* and adjacent genera reconstructed by Bayesian inference and maximum likelihood methods based on whole mitochondrial genome sequences, node values show posterior probabilities/bootstrap

A total of 32 whole mitochondrial genome sequences were included in the phylogenetic analysis. Both BI and ML methods show a similar topology. In the BI tree, 25 out of 29 nodes within the ingroup possess posterior probabilities of 1.00, and the remaining four nodes possess posterior probabilities equal to, or higher than 0.80. In the ML tree, 18 out of 29 nodes possess bootstrap values of 100, and the remaining nodes are equal to, or higher than 76. The genus *Abbottina* represented by three *A. rivularis* sequences, forms a monophyletic group itself, which is sister to the group formed by the genus *Pseudogobio* and the *Biwia*-*Microphysogobio* complex (Fig. 7). *Abbottina lalinensis* (ASIZB-WFD01LN) was nested within the genus *Microphysogobio*, and it is sister to the group formed by *M. springeri* and *M. yaluensis*, with nodal values of 0.97 (BI) and 86 (ML).

## Discussion

The controversy over the taxonomic status of *Abbottina* species primarily stems from the description of the generic characteristics (Yue 1998; He et al. 2017; Zhang et al. 2020). A detailed examination of the type species of the genus is crucial when the definition is ambiguous (Sun et al. 2021). *Abbottina rivularis* possesses several significant characters different from those seven genera of the *Biwia*-*Microphysogobio* complex as follows: (1) jaws concealed beneath lips, without horny margin (vs. jaws exposed, possessing horny margin, Fig. 3B, except the genus *Biwia*); (2) lower lip with anterior fold (vs. without anterior fold, Fig. 3B, except the genus *Biwia*); (3) anterior chamber of air-bladder encapsulated in thin soft membrane (vs. enclosed in fibrous capsule amongst all seven genera, Fig. 4B); (4) posterior chamber of air-bladder large (vs. small, Fig. 4B, except the genus *Biwia* Jordan & Fowler, 1903 and *Mesophysogobio* Sun, Tang & Zhao, 2025); (5) the lateral ethmoid broad, triangular-shaped (vs. narrow, crescent-shaped, Fig. 5B); (6) the jugal superiorly contacted with lateral ethmoid, with the junction length three-quarters in total length of upper jugal margin (vs. approximately one-eighth amongst all seven genera, Fig. 5B); and (7) the third infraorbital narrower than the jugal (vs. not narrower than the jugal amongst all seven genera, Fig. 5B). A summary on the diagnostic characters amongst the genus *Abbottina* and the genera within the *Biwia*-*Microphysogobio* complex is shown in Table 2.

**Table 2.** Morphological comparisons between the genus *Abbottina* and the seven genera within the *Biwia*-*Microphysogobio* complex

		* Abbottina *	* Biwia *	* Microphysogobio *	* Huigobio *
Mouth	(1) Horny sheaths on jaws	Absent	Absent	Present	Present
	(2) Papillae on lips	Less-developed	Less-developed	Developed	Developed
	(3) Anterior fold on lower lip	Present	Present	Absent	Absent
	(4) Barbels	Present	Absent	Present	Present
Orbit	(5) Junction between ethmoid and dorsal margin of jugal	Wide	Narrow	Narrow	Narrow
Air-bladder	(6) Anterior chamber encapsulation tissue	Membrane	Fibro	Fibro	Fibro
	(7) Posterior chamber length	Longer than anterior chamber	Longer than anterior chamber	Shorter than anterior chamber	Shorter than anterior chamber
		* Oriengobio *	* Mesophysogobio *	* Crossocheilogobio *	* Platysmacheilus *
Mouth	(1) Horny sheaths on jaws	Present	Present	Present	Present
	(2) Papillae on lips	Developed	Developed	Developed	Developed
	(3) Anterior fold on lower lip	Absent	Absent	Absent	Absent
	(4) Barbels	Present	Present	Present	Present
Orbit	(5) Infraorbital plate(s) connected with prefrontal bone	Narrow	Narrow	Narrow	Narrow
Air-bladder	(6) Anterior chamber encapsulation tissue	Fibro	Fibro	Fibro	Fibro
	(7) Posterior chamber length	Usually Shorter than anterior chamber	Longer than anterior chamber	Shorter than anterior chamber	Shorter than anterior chamber

*Abbottina lalinensis* was originally described by Huang and Li (1987) based on seven specimens from the Lalinhe River in Wuchang County, Heilongjiang Province. He et al. (2017) revised this species but still retained it under the genus *Abbottina*. According to the original description (Huang and Li 1987) and the specimens we collected, *A. lalinensis* should be placed in the genus *Microphysogobio* Mori, 1934 based on four morphological characters: (1) horny margin present on the jaws, and upper-jaw horny margin larger than half mouth width (Fig. 3C); (2) the lower lip without anterior fold of this species (Fig. 3C); (3) lateral lobes on lower lips not in contact with each other behind medial pad (Fig. 3C); and (4) jugal superiorly contacted with lateral ethmoid with narrow junction (Fig. 5C). The molecular phylogenetic trees reconstructed in this study based on whole mitochondrial genome sequences show that *A. lalinensis* was nested within the genus *Microphysogobio* (Fig. 7). The node values of the branch comprising *A. lalinensis*, *M. springeri*, and *M. yaluensis* are also relatively high. This result is consistent with the previous study based on Cyt *b* and nuclear loci (He et al. 2017). Both morphological and molecular phylogenetic evidence support *A. lalinensis* as a *Microphysogobio* species.

*Abbottina binhi* Nguyen, 2002 was described from the Bàng River in Cao Bàng Province, northern Vietnam. According to the original description, distribution, and specimens collected and examined from Cao Bàng Province, we assign this species to *Oriengobio* Sun, Tang & Zhao, 2025 based on the following characters: (1) jaws exposed (vs. covered with lips in *Abbottina*); (2) anterior fold on lower lip absent (vs. present in *Abbottina*); (3) branched anal-fin rays six (vs. five in *Abbottina*); and (4) scales above lateral line 3.5 (vs. 5.5–6.5 in *Abbottina*).

## Materials examined

***Abbottina rivularis***: A total of 67 specimens examined. • **ASIZB 186008–17**, ten specimens, 55.4–66.9 mm SL, from the Baihe River in the Haihe River basin, Hou’anling Village, Tanghekou Township, Huairou District, Beijing (c. 40.67198088°N, 116.66605228°E, 257 m a.s.l.); 30 July 2009. • **ASIZB 231709–10**, two specimens, 97.8–99.0 mm SL, from the Jiuduhe River in the Haihe River basin, Jiuduhe Township, Huairou District, Beijing (c. 40.41238905°N, 116.34811858°E, 240 m a.s.l.); 21 April 2022. • **ASIZB 248569**, one specimen, 79.3 mm SL, from the Liulimiaohe River in the Haihe River basin, Liulimiao Township, Huairou District, Beijing (c. 40.61430685°N, 116.61901901°E, 346 m a.s.l.); 12 May 2025. • **ASIZB 248049–54**, six specimens, 56.7–83.8 mm SL, from the Huaishahe River in the Haihe River basin, Bohai Township, Huairou District, Beijing (c. 40.41711235°N, 116.45071307°E, 212 m a.s.l.); 27 January 2024. • **ASIZB 197096–9**, four specimens, 48.5–73.7 mm SL, from the Qingshuihe River in the Haihe River basin, Taishitun Township, Miyun District, Beijing (c. 40.53093427°N, 117.12773883°E, 166 m a.s.l.); 6 July 2013. • **ASIZB 195237–9**, three specimens, 59.4–68.8 mm SL, from the Yongdinghe River in the Haihe River basin, Yanchi Township, Mentougou District, Beijing (c. 40.01674711°N, 115.82768621°E, 276 m a.s.l.); 24 May 2012. • **ASIZB 197161–3**, three specimens, 54.6–76.6 mm SL, from the Andamuhe River in the Haihe River basin, Xinchengzi Township, Miyun District, Beijing (c. 40.65000621°N, 117.43000804°E, 504 m a.s.l.); 7 July 2013. • **ASIZB 186007**, one specimen, 68.3 mm SL, from the Jinhaihu Lake in the Haihe River basin, Jinhaihu Township, Pinggu District, Beijing (c. 40.18484788°N, 117.30254270°E, 88 m a.s.l.); 9 October 2009. • **ASIZB 208485–7**, three specimens, 54.3–62.1 mm SL, from the Juhe River in the Haihe River basin, Donggaocun Township, Pinggu District, Beijing (c. 40.10569071°N, 117.06993776°E, 25 m a.s.l.); 24 April 2018. • **ASIZB 227942, 227948–51, 227953–4**, seven specimens, 59.0–82.9 mm SL, from the Jinhaihu Lake in the Haihe River basin, Jinhaihu Township, Pinggu District, Beijing (c. 40.18244437°N, 117.33318184°E, 106 m a.s.l.); 25 May 2021. • **ASIZB 233197–202**, six specimens, 56.2–94.2 mm SL, from the Yongdinghe Diversion Canal in the Haihe River basin, Yangfangdian Sub-district, Haidian District, Beijing (c. 39.91533737°N, 116.30957925°E, 52 m a.s.l.); 10 November 2022. • **ASIZB 219128–9**, two specimens, 66.6–77.9 mm SL, from the Huaihe River in the Haihe River basin, Tumen Township, Zanhuang County, Shijiazhuang City, Hebei Province (c. 37.62442182°N, 114.22879080°E, 264 m a.s.l.); 1 September 2020. • **ASIZB 219140**, one specimen, 77.6 mm SL, from the Liuhe in the Luanhe River basin, Lijiaying Township, Xinglong County, Chengde City, Hebei Province (c. 40.63447654°N, 117.77883266°E, 395 m a.s.l.); 27 September 2020. • **ASIZB 244624–5**, two specimens, 83.2–91.3 mm SL, from the Yellow River, Tianpingfeng Township, Pianguan County, Xinzhou City, Shanxi Province (c. 39.49612501°N, 111.41495403°E, 896 m a.s.l.); 25 May 2023. • **ASIZB 244125–8**, four specimens, 62.5–86.0 mm SL, from the Luanhe River, Xiangtang Sub-district, Luanzhou City, Tangshan City, Hebei Province (c. 39.72040580°N, 118.78110720°E, 24 m a.s.l.); 11 February 2023. • **ASIZB 225484–5**, two specimens, 64.3–76.4 mm SL, from the Huanghe River in the Huaihe River basin, Yanhe Township, Guangshan County, Xinyang City, Henan Province (c. 31.77607848°N, 114.81306128°E, 57 m a.s.l.); 23 April 2021. • **ASIZB 248048**, one specimen, 67.4 mm SL, from the Taohe in the Haihe River basin, Jucheng Township, Pingding County, Yangquan City, Shanxi Province (c. 37.88485574°N, 113.81419755°E, 477 m a.s.l.); 27 July 2024. • **ASIZB 248046–7**, two specimens, 55.4–73.7 mm SL, from the Nianhe River in the Yellow River basin, Niangzishen Township, Jingle County, Xinzhou City, Shanxi Province (c. 38.34517064°N, 112.04447117°E, 1277 m a.s.l.); 16 May 2024. • **ASIZB 246214–5**, two specimens, 33.2–39.2 mm SL, from the Fu’erjiang River in the Yalu River basin, Fujiang Township, Tonghua County, Tonghua City, Jilin Province (c. 41.77665171°N, 125.32282908°E, 433 m a.s.l.); 19 August 2023. • **ASIZB 248141–5**, five specimens, 30.7–47.8 mm SL, from the Qijiahe River in the Yellow River basin, Qijiahe Township, Xiaxian County, Yuncheng City, Shanxi Province (c. 34.94552005°N, 111.59968135°E, 322 m a.s.l.); 17 July 2024.

***Microphysogobio hsinglungshanensis***: A total of 22 specimens examined. • **ASIZB 240783–9, 247902–5**, 11 specimens, 39.9–54.0 mm SL, from the Liuhe River in the Luanhe River basin, Lijiaying Township, Xinglong County, Chengde City, Hebei Province (c. 40.63447654°N, 117.77883266°E, 395 m a.s.l.); 27 September 2020. • **ASIZB 247940–50**, 11 specimens, 36.9–53.2 mm SL, from the Xinluanhe River in the Luanhe River basin, Bencheng Township, Luannan County, Tangshan City, Hebei Province (c. 39.55228893°N, 118.73085124°E, 16 m a.s.l.); 12 July 2023.

***Microphysogobio lalinensis***: A total of 27 specimens examined. • **ASIZB 248001–15**, 15 specimens, 47.3–57.8 mm SL, from the Shahe River, Yuantai Township, Wafangdian City, Dalian City, Liaoning Province (c. 39.62330850°N, 122.06705087°E, 45 m a.s.l.); 16 March 2021. • **ASIZB 247998–8000**, three specimens, 53.8–57.4 mm SL, from the Wudaogouhe River in the Yalu River basin, Sidaogou Township, Linjiang City, Baishan City, Jilin Province (c. 40.72151103°N, 124.37807337°E, 104 m a.s.l.); 20 August 2023. • **ASIZB 246441–4**, four specimens, 51.8–61.7 mm SL, from a tributary of the Aihe River in the Yalu River basin, Shicheng Township, Fengcheng City, Dandong City, Liaoning Province (c. 40.72151103°N, 124.37807337°E, 104 m a.s.l.); 15 August 2023. • **ASIZB 248035**, one specimen, 58.4 mm SL, from a tributary of the Caohe River in the Yalu River basin, Jiguanshan Township, Fengcheng City, Dandong City, Liaoning Province (c. 40.59630459°N, 124.08965295°E, 85 m a.s.l.); 16 August 2023. • **ASIZB 248026–7**, two specimens, 42.0–42.9 mm SL, from a tributary of the Aihe River in the Yalu River basin, Bianmen Township, Fengcheng City, Dandong City, Liaoning Province (c. 40.34584490°N, 124.10122133°E, 65 m a.s.l.); 17 August 2023. • **ASIZB 248024–5**, two specimens, 39.6–43.1 mm SL, from the Fu’erjiang River in the Yalu River basin, Wangqingmen Township, Xinbin Manchu Autonomous County, Fushun City, Jilin Province (c. 41.65927862°N, 125.31311183°E, 394 m a.s.l.); 19 August 2023.

## Supplementary Material

XML Treatment for Abbottina

## Additional information

### Artificial Intelligence (AI) use

The authors accept full responsibility for the content of the manuscript, including the disclosure of any use of AI.

No AI tools were used in the preparation of this manuscript.

### Author ORCIDs

Z. Sun https://orcid.org/0000-0003-0565-2801

D. Sheng https://orcid.org/0009-0007-2515-8655

W. Tang https://orcid.org/0000-0001-5992-5022

Y. Zhao https://orcid.org/0000-0002-4615-596X

### Data availability

All of the data that support the findings of this study are available in the main text or Supplementary Information.

## Supplementary materials

Supplementary material 1 Supplementary table SS1 Data type xlsx Explanation note Detailed information of the mitochondrial genome sequences of *Abbottina* species and closely related species for molecular analysis. https://binary.pensoft.net/file/1625217This dataset is made available under the Open Database License (http://opendatacommons.org/licenses/odbl/1.0/). The Open Database License (ODbL) is a license agreement intended to allow users to freely share, modify, and use this Dataset while maintaining this same freedom for others, provided that the original source and author(s) are credited. Zhixian Sun, Dong Sheng, Shuya Sun, Wenqiao Tang, Yahui Zhao
